# Genomic diversity in ochratoxigenic and non ochratoxigenic strains of *Aspergillus carbonarius*

**DOI:** 10.1038/s41598-018-23802-8

**Published:** 2018-04-03

**Authors:** Gemma Castellá, M. Rosa Bragulat, Laura Puig, Walter Sanseverino, F. Javier Cabañes

**Affiliations:** 1 Veterinary Mycology Group, Department of Animal Health and Anatomy, Universitat Autònoma de Barcelona, Bellaterra, Catalonia Spain; 2 Sequentia Biotech SL, Barcelona, Catalonia Spain

## Abstract

Ochratoxin A (OTA) is a mycotoxin with nephrotoxic effects on animals and humans. *Aspergillus carbonarius* is the main responsible for OTA contamination of grapes and derived products. We present the genome resequencing of four *A. carbonarius* strains, one OTA producer and three atypical and unique non-OTA producing strains. These strains were sequenced using Illumina technology and compared with a reference genome of this species. We performed some specific bioinformatics analyses in genes involved in OTA biosynthesis. Data obtained in this study revealed the high genomic diversity within *A. carbonarius* strains. Although some gaps of more than 1,000 bp were identified in non-ochratoxigenic strains, no large deletions in functional genes related with OTA production were found. Moreover, the expression of five genes of the putative OTA biosynthetic cluster was down regulated under OTA-inducing conditions in the non-ochratoxigenic strains. Knowledge of the regulatory mechanisms involved in OTA biosynthesis will provide a deeper understanding of these non-ochratoxigenic strains.

## Introduction

Ochratoxin A (OTA) is a potent nephrotoxin which is found mainly in cereals and their products, but it also occurs in a variety of common foods and beverages such chocolate, dried fruits, coffee or wine. Besides this toxin is also a potent renal carcinogen in rodents and a possible cancer threat to humans. It is classified as a group 2B carcinogen by the International Agency for Research on Cancer^1^.

This mycotoxin is produced by several species of *Penicillium* and *Aspergillus* among which *Aspergillus carbonarius* is the main responsible source of this mycotoxin in wine or dried vine fruits from main viticultural regions worldwide^2,3^. The chemical structure of OTA consists of a polyketide derived chlorinated-dihydromethyl-isocoumarin moiety linked to phenylalanine by an amide bond. However, little is known about the genes involved in the OTA biosynthesis of the ochratoxigenic species. To date, OTA biosynthetic studies have only examined the inactivation of one gene and/or measure the expression of other genes encoded within an OTA gene cluster to investigate a correlation with its production^4–12^. No study has been conducted where each gene in an identified OTA gene cluster was knocked out. It is not yet clear which genes, or protein domains, found within an OTA biosynthetic gene cluster are essential for its production^13^.

So far only some OTA related PKS genes have been detected in OTA producing species such as *Aspergillus ochraceus, Aspergillus westerdijkiae, Penicillium nordicum, Penicillium verrucosum, Aspergillus niger* and *Aspergillus steynii*^4–8,11,14–16^. Functional characterizations of some PKS and NRPS genes have been performed by gene inactivation and expression experiments in *P. nordicum*^5,6^, *P. verrucosum*^7,11^, *A. ochraceus*^4^, *A. westerdijkiae*^8^, and *A. niger*^16^.

In *A*. *carbonarius*, the reference genome sequence of the OTA producer strain ITEM 5010 (Acv3), which was generated by the United States Department of Energy’s Joint Genome Institute (http://jgi.doe.gov/carbonarius/)^17,18^, has been helpful in the study of genes potentially involved in OTA biosynthesis^10,12,19–21^. Up to now, a nonribosomal peptide synthetase (*AcOTAnrps*) gene^10^, a polyketide synthase (*AcOTApks*) gene^12^ and a halogenase gene (*AcOTAhal*)^20^ have been related to the OTA biosynthetic gene cluster in this species.

Mycotoxin production consistency varies in the reported mycotoxigenic species. For example, a high percentage of nonaflatoxigenic isolates are present in some *Aspergillus flavus* populations^22^. In the same way, most of the isolates of *A. niger* are not able to produce OTA^23^. On the contrary, nearly 100% of the isolates of *A. carbonarius* produce OTA and its production is a very consistent property when monitored on CYA agar^24^.

In nonaflatoxigenic isolates of *A. flavus* deletion of a part or the entire aflatoxin gene cluster is not rare and the resulting deletion patterns are diverse and affect different coding regions^25^. Similarly, in the non ochratoxigenic *A. niger* strains ATCC 1015 and ATCC 9029^26^, a 21-kb deletion in a remnant of the PKS gene (An15g07920) of the putative ochratoxin cluster in the ochratoxigenic strain *A. niger* CBS 513.88^27^ was identified.

Recently, we used the Ion Torrent technology to resequence the genome of an atoxigenic wild strain of *A. carbonarius*^19^. We detected that in the atoxigenic strain there was a high accumulation of nonsense and missense mutations in PKS and NRPS encoding genes. The high mutation rate of these genes could explain the lack of production of OTA by the atoxigenic strain. A full characterization of the gene clusters responsible for OTA production in these species will show whether all isolates in any of the species reported to produce OTA actually have the gene cluster required. On the other hand, the inability to produce OTA may also be caused by silent genes or by mutations in functional or regulatory genes^28^.

Here we present the genome resequencing of four *A. carbonarius* strains, one OTA producer and three atypical non-OTA producing strains. In the present work, these strains were sequenced using Illumina technology and compared with the genome reference Acv3. Besides this main objective, and due to the fact that three of these strains do not produce OTA, we performed some new specific bioinformatics analyses in genes involved in OTA biosynthesis. We focused these analyses on nonsense and missense mutation detection, and also in to identify whether large DNA sections of the reference genome Acv3 or of the new sequenced OTA producing strain were absent in the genome of the three non-OTA producing strains.

## Results

### OTA production in the *A. carbonarius* strains studied

*Aspergillus carbonarius* strains grew in CYA medium at 15 °C, 25 °C and 30 °C. All strains presented good growth with proper sporulation forming typical black colonies. *A. carbonarius* ITEM 5010 (=A-1796) and A-1137 produced OTA at detectable levels at the three temperatures of incubation tested (Table 1). Strain ITEM 5010 produced higher amounts of OTA than strain A-1137 at 15 °C after 10 and 30 days of incubation, whereas maximum OTA production in strain A-1137 was at 25 °C. The non-OTA producing strains (A-2160, A-2579 and A-2594) were not able to produce OTA at these temperatures after 3, 10 or 30 days of incubation.

**Table 1:** Ochratoxin A concentration (mean value in µg/g) detected in the 5 strains of *A. carbonarius* in CYA at each temperature and incubation time tested.

Strain	Temperature	OTA (in µg/g)								
		15 °C			25 °C			30 °C		
	days	3	10	30	3	10	30	3	10	30
A-1137		ND	29.8	37.07	40.14	61.8	64.48	38.54	53.52	54.64
A-1796*		ND	197.12	190.06	34.66	7.08	6.80	1.18	0.38	0.39
A-2160		ND	ND	ND	ND	ND	ND	ND	ND	ND
A-2579		ND	ND	ND	ND	ND	ND	ND	ND	ND
A-2594		ND	ND	ND	ND	ND	ND	ND	ND	ND

ND, not detected, Limit of quantification: 0.06 µg/g.

*Strain ITEM 5010.

**Figure 1:**
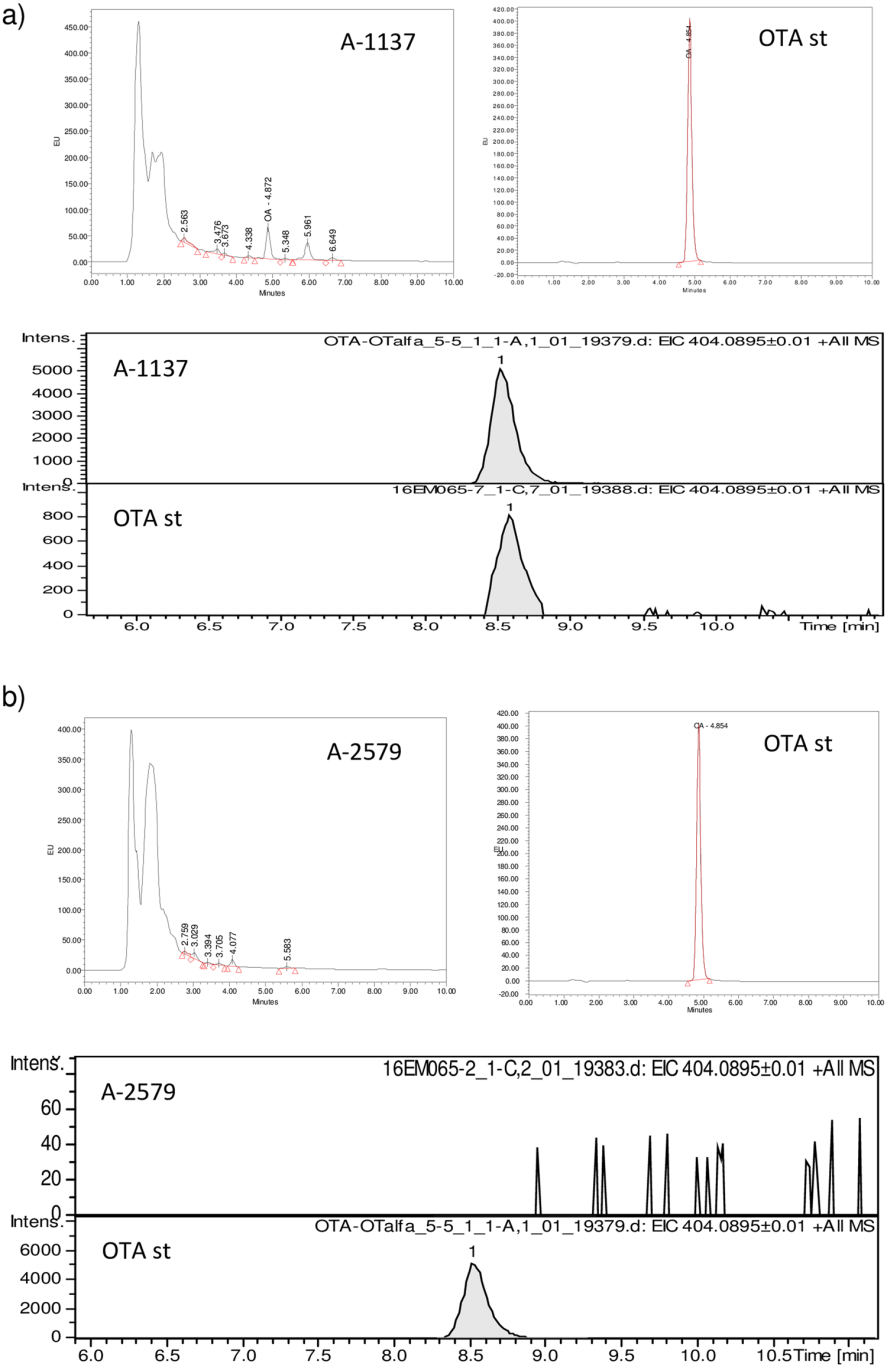
HPLC-FLD chromatograms and mass spectra of (**a**) the OTA producing strain *A. carbonarius* A-1137 (OTA standard retention time: 4.854 min), and (**b**) the non-ochratoxigenic strain of *A. carbonarius* A-2579 after incubation at 15 °C for 10 days on Czapek Yeast extract Agar.

The identity of OTA was confirmed by HPLC-MS. Figure 1 shows some selected chromatograms and mass spectra of the fungal strains analyzed in this study. Extracts of *A. carbonarius* A-1137 (Fig. 1a) presented a clear peak with the same retention time (4.7 minutes) and mass spectrum of OTA. The extracts of *A. carbonarius* A-2160, A-2579 (Fig. 1b) and A-2594 showed no signals at the same retention time of OTA.

### Resequencing study

A summary of the general resequencing genome data of the four *A. carbonarius* strains is shown in Table 2. More than 95 millions (M) of paired-end (PE) reads, for a total of 14.25 Gb of sequence data, with an insert size of 500 bp was generated using an Illumina HiSeq. 1500 sequencer. A mean of 85% of reads passed the filtering and trimming quality step and for all the four strains it was possible to have a breath coverage of 84% and the depth coverage was between 35.06x and 68.77x. A range between 10.6 to 17.6% of unmapped reads was produced. The mapping of the reads from the four strains against the reference genome Acv3 was the starting point to study the variome and to highlight the genomic differences that might explain the phenotype of the atoxigenic strains.

**Table 2:** Statistics of resequencing results.

	A-1137	A-2160	A-2579	A-2594
Number of reads before trimming	20,201,150	30,051,642	19,157,966	23,126,354
Number of reads after trimming	17,509,717	26,271,660	14,516,307	17,815,222
Percentage of reads GC content alter trimming	50%	50%	48%	51%
Percentage of aligned reads	87.2%	86.3%	82.4%	89.4%
Percentage of Q30 aligned reads	86.8%	85.9%	82%	89.3%
Percentage of unmapped reads	12.8%	13.7%	17.6%	10.6%
Aligned reads without duplications	7,808,891	11,567,359	6,118,604	8,133,583
Number of high confidence reads	7,349,270	10,803,126	5,717,306	7,643,479
Bases Mapped	30,005,888	30,382,422	30,413,427	30,356,751
Mean depth of coverage	48.00	68.77	35.06	36.70
Coverage of genome (%)	83%	84%	84%	84%

### SNPs and DIPs analysis

A total amount of 226,930, 55,215, 37,307 and 37,322 variants were identified for A-1137, A-2160, A-2579 and A-2594, respectively (Table 3). As can be observed, the sample A-1137, which is the ochratoxigenic strain, shows a significantly higher number of variants respect to the others, having a 7.05 variants/KB. Furthermore, it is important to mention that most of the detected variants have a homozygous genotype. On the other hand, private and shared variants were identified. Focusing in non-ochratoxigenic strains, about 7,000 common variants not present in A-1137 were detected.

**Table 3:** Summary of SNP and DIP analysis.

Variant Calling (hard filtering) SNP/DIP	A-1137	A-2160	A-2579	A-2594
Depth	47,401	65,3801	35,3255	27,5058
Total variant positions	265,360			
Total variants	226,930	55,215	37,307	37,322
Mean variants/KB	7,05336	1,80396	1,2174	1,21708
Heterozygous variants	2,498	1,785	1,580	1,500
Homozygous variants	224,432	53,430	35,727	35,822
Heterozygous private variants	1,205	232	83	193
Homozygous private variants	189,131	19,954	3	11
Common homozygous ALT variants	9,038			
Common heterozygous ALT variants	511			
Common homozygous ALT variants (not present in A1137)	7,344			
Common heterozygous ALT variants (not present in A1137)	161			

As one of our objectives was to find differences among the ochratoxigenic and the non-ochratoxigenic strains, we focused our analysis in some genes potentially involved in OTA biosynthesis. We selected a total of 146 genes which have been described to be up-regulated under OTA induction conditions in a recent transcriptome analysis of four OTA producing strains^21^. We also analyzed in depth five genes located in the hypothetical OTA biosynthetic gene cluster 38 in scaffold 12. Using bedtools utility and SnpEff annotations, the variants affecting to this group of genes were extracted, having a total number of 3,678 variants. The most relevant variants (911 variants), attending to the severity of their effect on the gene product, were retrieved (Table 4). Above them, 704 and 34 variants turned out to be private for A-1137 and A-2610, respectively. We found no private variants for A-2579 and A-2594 samples. In addition, 40 variants were classified as common for all the strains, and 17 common variants only for the strains without OTA production. These variants affected to different genes (Table 5). Among these genes, a cytochrome P450 monooxygenase and a PKS were affected but none of them were located in scaffold 12.

**Table 4:** Summary of SNP and DIP analysis in selected genes.

Annotation	Relevant SNPs	Private A-1137	Private A-2160	Common to all strains	Common to atoxigenic strains
disruptive_inframe_deletion	1	0	0	0	0
disruptive_inframe_insertion	1	1	0	0	0
frameshift_variant	6	4	1	0	0
inframe_deletion	5	4	1	0	0
inframe_insertion	3	2	0	0	0
missense_variant	879	682	31	38	17
missense_variant&splice_region_variant	3	2	0	1	0
start_lost&disruptive_inframe_insertion	1	1	0	0	0
stop_gained	10	8	1	0	0
stop_lost&splice_region_variant	1	0	0	0	0
stop_retained_variant	1	0	0	1	0
Total	911	704	34	40	17

**Table 5:** Genes with common variants in atoxigenic strains.

Protein ID	Functional category
39367	**Dynamin family protein-** e_gw1.1.862.1 (scaffold 1)
156387	**NACHT domain protein-** estExt_Genewise1.C_230145 (scaffold 23)
6644	**Matrix metalloproteinase**-11- Genemark1.6644_g (scaffold 9)
131073	**O-methyltransferase** (MT)- estExt_Genemark1.C_80577 (scaffold 8)
492	**O-methyltransferase** (MT)- Genemark1.492_g (scaffold 1)
208126	**Cytochrome P450 monooxygenase** (CYP) fgenesh_isotigs_kg.8_#_533_#_isotig12280 (scaffold 8)
504341	**O-methyltransferase** (MT) estExt_fgenesh2_pg.C_3_t10258 (scaffold 3)
507488	**Proline oxidase** (Put1) estExt_fgenesh2_pg.C_90064 (scaffold 9)
206969	**N-acetyltransferase** fgenesh_isotigs_kg.6_#_549_#_isotig11985 (scaffold 6)
56260	**PKS-** e_gw1.16.5.1 (scaffold 16)

Regarding the five genes located in the hypothetical OTA biosynthetic gene cluster, we found 34 missense variants and 1 stop gained mutation in *AcOTApks* gene. Only 5 missense variant mutations were private of non-ochratoxigneic strains. In *AcOTAnrps* gene, 70 missense variants and 1 stop gained mutations were found and none of them were private of non-ochratoxigenic strains. In *AcOTAhal* gene only one missense variant was detected in strain A-2160. In *AcOTAp450* and *AcOTAbZIP* mutations were detected only in the ochratoxigenic strain A-1137. (Supplementary Table 1).

### CNV and SV analysis

Using a read depth approach on the multiple mapping alignment, five major gaps of more than 1,000 bp were identified in non-ochratoxigenic strains. Three of them were present in all non-ochratoxigenic strains. These gaps affected to 1) estExt_Genemark1.C_60265 gene, a negative modulator of initiation of replication SeqA; 2) e_gw1.9.287.1 gene, a member of tautomerase/MIF superfamily; 3) estExt_Genemark1.C_90522 gene, a ribosomal protein L13 (structural constituent of the ribosome).

### Expression analysis of OTA genes in *A. carbonarius*

**Figure 2:**
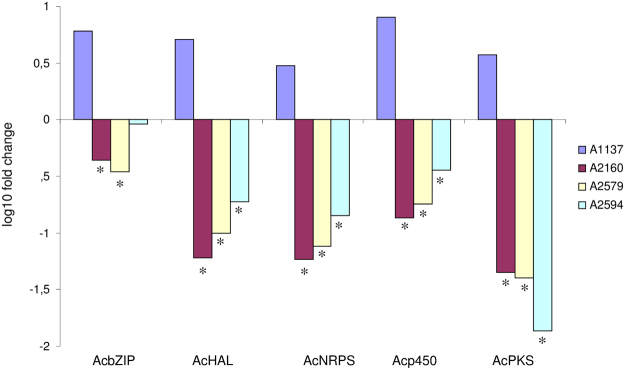
Relative expression analysis by real time PCR of *AcOTApks*, *AcOTAnrps*, *AcOTAhal*, *AcOTAbZIP* and *AcOTAp450* genes in *A. carbonarius* strains A-1137, A-2160, A2579 and A-2594 grown on Czapek Yeast extract broth. (*P < 0.05).

We studied the transcription profiles of the three biosynthetic genes characterized so far (*AcOTApks*, *AcOTAnrps*, and *AcOTAhal*) and two genes located next to them in the same cluster, *AcOTAp450* and *AcOTAbZIP*. Two genes, β-tubulin and ubiquitin, were used as reference genes. OTA was detected in both ochratoxigenic strains ITEM 5010 (0.77 ± 0.296 μg/g) and A-1137 (10.69 ± 1.672 μg/g) whereas it was not detectable in non-OTA producing strains. From the relative gene expression analysis, it was observed that transcription of all the genes in the non-ochratoxigenic strains was significantly down-regulated compared to both OTA-producing strain (Fig. 2).

## Discussion

In the present study, the genome of four *A. carbonarius* strains was successively resequenced using the Illumina HiSeq. 1500 platform. One of the strains was an OTA-producer and the rest were non-OTA producers. Due to the fact that non-ochratoxigenic isolates of *A. carbonarius* are very rarely found in nature, their inability to produce OTA was tested at different culture conditions and posteriorly confirmed by HPLC-MS. In general, we should consider that a mycotoxin producing species should produce consistently a mycotoxin and not only traces. In our study we have not to detect a very low quantity of a mycotoxin in a food; we have to detect and define a mycotoxin producer, that, in theory produce an easy detectable quantity of this product. Besides, a weak production should be clearly distinguishable from no production^2^. We consider positive strains as consistent producers rather than ones that may only produce trace amounts, which may be very low mycotoxin producers. However, in our study, none of the non OTA producing strains were able to produce OTA in any of the temperature and incubation times tested. Moreover, in order to confirm these results, the extracts were analyzed by HPLC-MS and the non producers showed no traces of OTA in any condition tested. In addition, these strains had been checked also in different conducive conditions and at 60 and 120 days of incubation and no OTA was detected by HPLC-MS^29^.

We performed a comparison among them and the reference genome Acv3 of the same species^17,18^. One of the strains, A-2160, has been resequenced before by our group using Ion Torrent platform^19^. Data comparison between IonTorrent and Illumina show more robust results with Illumina (lower error ratio) although the coverage of the genome was similar.

Data presented in this study emphasize the high genomic diversity present within *A. carbonarius* strains. This intraspecific variation has also been demonstrated in OTA production^24^. Our comparative genomic study revealed that the OTA-producing strain has more mutations than the non-ochratoxigenic strains. As more *A. carbonarius* genomes are sequenced and made publicly available, probably we will uncover more genomic variation. In a recent article a great within-species variation in secondary metabolic gene clusters was revealed when 66 genomes of *Aspergillus fumigatus*, most of them (58) retrieved from public database, were compared^30^.

**Figure 3:**
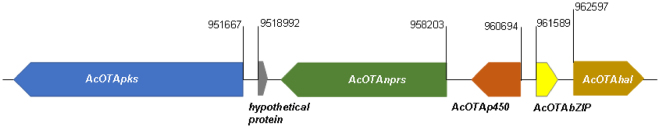
Schematic representation of the putative OTA biosynthetic gene cluster 38, located on scaffold 12 (931076-971429 nt). Arrows represent genes and point in the direction of transcription. The role of the polyketide synthase gene (*AcOTApks*), the non-ribosomal peptide synthetase gene (*AcOTAnrps*) and the halogenase gene (*AcOTAhal*) in the biosynthetic pathway of OTA has been determined by gene inactivation^10,12,20^. The cytochrome P450 monooxygenase gene (*AcOTAp450*) and the bzip transcription factor gene (*AcOTAbZip*) are putatively involved in OTA biosynthesis^20,21^.

In general, the biosynthesis genes for fungal secondary metabolites in *Aspergillus* are located in physical proximity to each other, forming gene clusters that usually harbor genes for PKS, NRPS, hydrolases, oxidases, methylases, transporters, and regulatory proteins^31^. The availability of the genome sequence of *A. niger* and *A. carbonarius* allowed the experimental characterization of an OTA-cluster in both species (50–90% synteny)^18^. Recently, Gerin *et al*.^21^ studied the gene transcriptional profile of 4 ochratoxigenic *A. carbonarius* strains under inducing and non-inducing conditions for OTA production. The comparative transcriptome analysis of *A. carbonarius* strains showed 146 highly up-regulated genes under OTA inducing conditions and allowed the characterization of an hypothetical OTA gene cluster (cluster 38) in this species^21^. This cluster contains three genes, *AcOTApks, AcOTAnrps* and *AcOTAhal*, directly related to OTA biosynthesis^10,12,20^ and two other genes located in the same genomic region which could play a role in the biosynthesis pathway as part of the OTA cluster (Fig. 3). These genes were *AcOTAp450*, a cytochrome p450 monooxygenase, and *AcOTAbZIP*, a transcription factor which regulate multiple metabolic processes other than stress response, development, and morphology in *Aspergillus* species^32^.

Comparison of the sequences of the 146 genes highly up-regulated in OTA inducing conditions^21^, revealed that ochratoxigenic strain A-1137 showed more mutations than the rest of the strains. Non-ochratoxigenic strains showed 17 common missense variants located in different genes, mainly genes related with transferase activity and metabolic processes of carbohydrates, lipids, aminoacids, proteins and other macromolecules. None of these genes were located in the cluster 38, the hypothetical gene cluster of OTA in *A. carbonarius*^21^. When we focus on cluster 38 and the genes potentially involved in OTA biosynthesis, the OTA-producer strain showed variants in *AcOTApks*, *AcOTAnrps*, *AcOTAbZIP* and *AcOTAp450* but these genes were functional and their expression was similar to *A. carbonarius* ITEM5010. In atoxigenic strains only five common missense variants in *AcOTApks* gene were found. The CNV and SV analysis showed three common gaps in atoxigenic strains but none of them affecting to OTA cluster genes. Despite some studies have been carried out on molecular aspects of OTA biosynthesis, the length and composition of OTA cluster remain not completely defined. The key enzymes (*AcOTApks, AcOTAnrps* and *AcOTAhal*) and two other genes located in the same genomic region (*AcOTAp450* and *AcOTAbZIP*) which could play a role in the biosynthesis pathway as part of the OTA cluster have been described^10,12,20,21^. Other genes present in the same cluster, as the hypothetical protein (Fig. 3), need further investigation to establish their role in OTA cluster. Furthermore, it could be that two different clusters could be involved in OTA biosynthesis as it has been suggested in *A. westerdijkiae*^33^.

Even though no deletions in functional genes related with OTA production have been found in non-OTA producer strains, all these genes were under-regulated, including the transcription factor *AcOTAbZIP*. The lack of OTA production by these strains could be explained by regulatory mechanisms. Secondary metabolism gene regulation can be in part explained by transcriptional control through hierarchical levels of transcriptional regulatory elements, some of which are almost entirely specific for the respective pathway, and others of which display a more global regulation of secondary metabolism^34^. On the basis of the available genome information, about 60% of fungal secondary metabolism gene clusters contain a putative regulatory gene. Most of the potential regulators in fungal PKS-encoding gene clusters belong to the Zn cluster family of transcription factors, whereas NRPS-regulating transcription factors seem to be more diverse^35^. Some transcription factors has been described to be highly up-regulated under OTA conditions^21^, including a transcription factor of the Zn(2)-Cys(6) family. We analyzed their sequences and no mutations were found in non-ochratoxigenic strains.

In addition to cluster specific regulators, secondary metabolism gene clusters can also be regulated by broad domain transcription factors and heteromeric complexes which are encoded by genes that not belong to any cluster and also regulated a number of genes that are not involved in secondary metabolism. A well-studied example is the velvet complex VelB-VeA-LaeA^36^. Deletion of Lae in *Aspergillus nidulans* blocks the expression of metabolic gene cluster of sterigmatocystin biosynthesis^37^. This complex could have an important role regulating conidiation and OTA biosynthesis in response to light in *A. carbonarius*^38^. Crespo-Sempere *et al*.^38^ observed a strong reduction in conidiation and OTA production in VeA and LaeA deletion mutants. VeA and LaeA regulation might function at multiple layers of regulation network, such as transcription, post-transcriptional processing, translation, or posttranslational modification. In fact, LaeA can also act as a global regulator by influencing chromatin structure^35^. Regulation by chromatin modifications involving histone acetylation or methylation is of major importance for the modulation of fungal secondary metabolism^35^. In our study, the non-ochratoxigenic strains showed neither mutations nor deletions in the described LaeA protein of *A. carbonarius*.

Recently, genome resequencing has been useful for improving the knowledge on the regulation of sterigmatocystin biosynthesis in *Aspergillus nidulans*^39^. Mutants strains that were not able to produced sterigmatocystin were resequenced and causative mutations of this phenotype were characterized in 12 of 17 strains. In the remaining five strains, one strain showed no mutations and the structural variant analysis did not identify a credible variant. In four strains, some mutations were found in noncoding regions but they could not explain its phenotype by single gene knockout.

In conclusion, our study showed the genomic diversity present within *A. carbonarius* strains. The OTA producing strain showed a higher nucleotide variation than the three non-ochratoxigenic strains sequenced. Nucleotide variants in the OTA-producing strain were found in both genes up-regulated under OTA conditions and genes located in the hypothetical OTA cluster. More genetic variability will be observed as more genomes of *A. carbonarius* are available. The non-ochratoxigenic strains showed no deletions in functional genes related to OTA biosynthesis although all of them were down-regulated. It may be that regulatory mechanism of OTA biosynthesis could act within the biosynthetic cluster or external to it and its phenotypic expression is likely to subordinate to other regulatory processes acting at post-transcriptional level. However, the OTA cluster remains not completely defined. The analysis of the genomic data around this species will facilitate improved gene annotation and will be crucial for identifying regulatory elements. Further investigation of *A. carbonarius* transcriptome is needed to define the regulatory mechanisms involved in the biosynthesis of OTA in these non-ochratoxigenic strains.

## Methods

### OTA production ability detection

OTA production was confirmed using a previously described HPLC screening method designed in our laboratory^40^. The OTA-producing strains A-1137 and A-1796 (=ITEM 5010), and three non-OTA-producing strains of *A. carbonarius* A-2160, A-2579 and A-2594 from our fungal collection were first three point inoculated on Czapek Yeast extract Agar (CYA) and incubated at 15, 25 and 30 °C. After 3, 10 and 30 days of incubation at each temperature assayed and from each strain, three agar plugs were removed from different points of the colony and extracted with 0.5 ml of methanol. The extracts were filtered and maintained at 4 °C until their analysis. Four replicates for each isolate and incubation condition assayed were used. The entire experiment was repeated twice. In total, eight values of OTA for each isolate and incubation condition tested were obtained.

OTA quantification was made by a Waters 2695 chromatograph with a fluorescence detector Waters 2475 (excitation wavelength: 330 nm/emission wavelength: 460 nm), and with a Sunfire C18 column, 150 × 4.6 mm, i.d., 3.5 µm. Twenty μl of each extract were applied. The mobile phase was acetonitril/water/acetic acid (57/41/2, v/v/v) eluted at a flow rate of 1 ml/min. The extracts with the same retention time as OTA (around 4.8 min), were considered positive. The limit of quantification of the HPLC technique with the extraction procedure was 0.06 μg/g for OTA. In addition, the identity of OTA was confirmed in some selected samples by HPLC-MS. A 1200RR HPLC (Agilent Technologies, Waldbronn, Germany) connected to a micrOTOF-Q mass spectrometer (Bruker Daltonics, Bremen, Germany) system was used for the detection of these metabolites. An acetonitril extraction aliquot of each sample was filtered using 0.22 µm MS PVDF Syringe Filter from Membrane Solutions (Bellevue, USA), just before injection. The analytes were separated on a 150 × 4.6 mm i.d., 3.5 µm, Sunfire column preceded by a 0.5 µm guard filter, using an isocratic analysis (20:80, 0.5% HAcO and 1 mM NH4AcO in H_2_O: 0.5% HAcO and 1 mM NH4AcO in MeOH) with a flow rate 0.5 mL/min^10^. The column temperature was 25 °C, and the injection volume was 20 µl. The mass spectrometer was operated in the positive mode, using an electrospray source. The analysis was focused in m/z = 50–1000, using capillary voltage 4800 V, nebulizer gas 3.5 Bar, Dry Gas 7.0 L/min, Dry Temp 210 °C, Ion Energy 5.0 eV, Collision Energy 7.0 eV, Collision Cell RF 170.0 Vpp, Transfer Time 65 µs and PrePulse Storage Time 8.0 µs. Data acquisition was performed with otofControl version 3.2 and HyStar version 3.2 softwares (Bruker Daltonics, Bremen, Germany) and data processing was performed with Bruker Compass DataAnalysis 4.2 software (Bruker Daltonics, Bremen, Germany). Peak identifications were achieved by comparing retention times and mass spectra of sample peaks with those of standards prepared in acetonitril. All the extracts were injected in a sequence where first and last injection was an OTA standard, to verify that the response was stable during the injection samples. A sample without signal for this analyte was spiked with OTA to verify that there was no suppression signal in sample analysis.

### Genomic DNA extraction

The strains of *A. carbonarius* were grown on malt extract broth medium in the dark at 25 °C for 48 h. Mycelium was recovered and grounded into fine powder using a mortar and pestle after brief nitrogen deep freezing. DNA extraction was carried as described previously^19^.

### Genomic DNA sequencing and alignment

Nextera DNA Library Preparation Kit (Illumina, Inc., San Diego, California, USA) was used to construct libraries for Illumina HiSeq 1500 sequencing with an insert size of 500 bp. The 150-bp paired-end sequencing of the four strains was done by GenomiX4life (Baronissi, Italy).

Prior to further analysis, a quality check (trimming) was performed on the raw sequencing data, removing low quality portions while preserving the longest high quality part of a NGS reads. The minimum length established was 35 bp and the quality score 30. The raw sequence data coming from the high-throughput sequencing pipelines were applied to the program FastQC v0.11.2^41^ for quality control of sequencing. FastQC was used before and after the filtering process to evaluate the quality of the raw reads. The filter and trimming processes were made with Trimmomatic v0.33^42^ with parameters set to LEADING: 25, TRAILING: 25, HEADCROP: 13, SLIDINGWINDOW: 28, MINLE: 35.

The high-quality reads obtained after the trimming were aligned against the *A. carbonarius* v3 reference genome (http://genome.jgi.doe.gov/Aspca3/Aspca3.home.html). To check the mapping quality of the alignment, *samstat* software^43^ was used. The resulting alignment file was pre-processed to make it adequate for variant calling analysis. The reads with a mapping quality less than 30 were removed using Samtools v1.2^44^. Removal of duplicate reads was performed with *Picard-tools* v1.9^45^. Re-mapping of sequence reads around insertion deletion polymorphisms was performed using the InDEL realigner of GATK toolkit^46^, as a recommended standard practice.

### SNPs and DIPs

Variant Calling experiment was performed on the four *A. carbonarius* strains. The workflow can be generally summarized as a two-step process with alignment of the data to a genome reference followed by subsequent genetic variant calling from the post-alignment data.

Variant calling has been performed by SUPER-W v 4^47^ available at superw.sequentiabiotech.com. SUPER-W is an open-source, dynamic and fast tool to analyse the variation data produced from the resequencing experiments. SUPER uses Samtools v1.2^44^ to call small variations (such as SNPs and DIPs) and Delly tool v.0.6.5^48^ for the SVs (including deletions, inversions, duplications and translocations). SNPs and DIPs filtering was first performed using SnpSift v3.6c^49^. This tool set works on Variant Calling File (vcf) format. The applied filter included a minimum depth of 10 reads, PHRED quality of 30 and a homozygous value 0.8 (QUAL > 30; DP ≥ 10; AF1 ≥ 0.8). The vcf files data were analyzed using vcf-compare (VCFtools v0.1.12b^50^) and R v3.1.1^51^ tools to highlight common and individual variations between the sequenced strains. A manual filter, using bash scripting, was applied to decrease the number of false positives found. The homozygote unique variations, for each strain, were filtered with AF > 0.8. Any variation found was analyzed by SnpEff v4.1b tool^52^. Finally a deep annotation of the variants has been performed categorizing each variant based on its relationship to coding sequences in the genome and how it may change the coding sequence and affect the gene product. SnpEff v4.1b tool^52^ was chosen as the best software to perform this analysis. The results were visualized using IGV v2.3.34^53^. The single variations were individually analyzed visualizing the change-related coordinates of every snapshot produced by IGV.

### CNV and SV analysis

Bam files resulting from the mapping with SUPER were analyzed in order to get a list of CNVs with CNVnator v0.3.2^54^. Bin size was set at 100 bp after several trials in order to have a ratio between average Read Depth (RD) and standard deviation near 4. Several filtering criteria were used for each sample to reduce the amount of false positive variants. Only variants with a p-value < 0.01, CNV size > 1 Kb and q0 < 0.5 were retained for further analysis. This criterion was applied in previous CNV analysis^55^. A final filtering step was done to compare atoxigenic and toxigenic strains. For this, we intersected all unique variants present in atoxigenic strains against variants present in the OTA-producing strain. Those variants present only in atoxigenic strains which does not have reciprocal overlaps greater than 50% with any variant in the OTA-producing strain are retained for further analysis. Moreover, using DELLY v0.6.5^48^, a structural variant (SV) discovery tool that integrates paired-end and split read analysis, we have detected all possible SVs in all four samples. The output has been filtered thanks to a tag added by this tool, which classify all variants according to its quality. If a putative variant have at least 3 reads support with mapping quality (QUAL >  = 20), it is considered as a high probable variant, therefore, retained for further analysis. To better highlight putative deleted or duplicated regions, we crossed CNV results with SV results to get a final list of variant candidates found in both analyses. For this purpose, first we intersected all unique filtered SVs results for atoxigenic strains against the OTA-producing strain filtered variants with a minimum 50% reciprocal overlap (the same done before with CNV results) to finally intersect CNVs and SVs only present in atoxigenic strains.

### Variant association with previous studies

From a previous transcriptome study about the expression changes in *A*. *carbonarius* strains associated with OTA production^21^, 146 genes were selected to be putative involved in the OTA production. An integrated table of all the variation found in the four samples related to these genes was created in order to find clue about the variation related to OTA production.

### RNA extraction and cDNA synthesis

For gene expression analyses, strains were grown in Czapek Yeast extract broth medium in the dark at 25 °C for 48 h without shaking. Each strain was grown in triplicate. OTA determination was done by HPLC as described previously.

The mycelium was removed from the plates and was stored frozen at −80 °C prior to total RNA extraction. Frozen mycelium (100 mg) was powdered in liquid nitrogen and total RNA was isolated using QIAzol lysis reagent (Qiagen, Madrid, Spain) and purified by using RNeasy Plant Mini Kit (Qiagen, Madrid, Spain), following the manufacturer’s protocol. The extracted RNA was treated with DNAse (DNAse I, amplification grade, Invitrogen, Carlsbad, CA, USA) to remove genomic DNA contamination from the samples. The amount and quality of total RNA was estimated by a Nanodrop 2000 spectrophotometer (Thermo Fisher Scientific Inc., Wilmington, Delaware, USA) and a Bioanalyzer 2100 (Agilent Technologies, Santa Clara, CA, USA). First-strand cDNA was synthesized using the high capacity cDNA reverse transcription kit (Applied Biosystems, Foster City, CA, USA). Priming was done with random hexamers. For each RNA sample, cDNA was synthesized twice. The cDNA samples were kept at −20 °C.

### Analysis of gene expression by RT-qPCR

The transcription profiles of five genes of the ocratoxin A biosynthesis cluster of *A. carbonarius* (*AcOTApks*, *AcOTAnrps*, *AcOTAhal*, *AcOTAp450* and *AcOTAbZIP*) and β-tubulin and ubiquitin-conjugating enzyme genes as reference genes, were analyzed in all strains by using real-time quantitative reverse transcription-PCR (qRT-PCR). The primer sets were retrieved from previous works with the exception of primer pair for *AcOTAnrps*. This primer pair was designed using the Primer Express software (Applied Biosystems, Foster City, CA, USA) and the nonribosomal peptide synthetase gene (ID 132610) involved in OTA biosynthesis^10^, according to MIQE guidelines^56^. The sequences of these primers used for real time PCR are shown in Supplementary Table 2.

Real time PCR were performed using an Applied Biosystems 7500 Real Time PCR system programmed to hold at 50 °C for 2 min, to hold at 95 °C for 10 min, and to complete 40 cycles of 95 °C for 15 s and 60 °C for 1 min. Real Time PCR reactions were performed using the PowerUp SYBR-Green PCR Master Mix (Applied Biosystems, Foster City, CA, USA) according to the recommendations of the manufacturer. Amplification mixtures for Real Time PCR reactions contained 2 μl of template cDNA and different concentrations of primers pairs for each gene were added (Supplementary Table 2) in a final volume of 25 μl. The RNA samples for each replication and cDNA synthesis were run in triplicate. The results were normalized using β-tubulin and ubiquitin-conjugating enzyme amplifications run on the same plate (endogenous controls). Data analysis was carried out using DataAssist software V.3 (Applied Biosystems, Foster City, CA, USA). Relative quantification of gene expression was calculated using the −2^ΔΔCt^ method and all data was normalized to β-tubulin and ubiquitin. Expression difference among strains was assessed for statistical significance using a pairwise fixed reallocation randomization test within the Relative Expression Software Tool v2.0.13 (REST 2009)^57^, taking the divergent efficiencies into account. The calibrator sample corresponded to the value of expression of the OTA- producing strain ITEM 5010.

### Accession codes

The genome resequencing information of *A. carbonarius* A-1137, A-2160, A-2579 and A-2594 have been deposited in the NCBI Sequence Read Archive (SRA) database under accession SRP125447.

## Electronic supplementary material

Supplementary information

## References

[1] Pfohl-Leszkowicz A, Manderville RA (2012). An update on direct genotoxicity as a molecular mechanism of ochratoxin a carcinogenicity. Chem. Res. Toxicol..

[2] Cabañes, F. J. & Bragulat, M. R. Ochratoxin A in profiling and speciation Aspergillus *in the Genomic Era*. Varga, J. & Samson, R. A. (ed.) 57–70 (Wageningen Academic Publishers, The Netherlands, 2008).

[3] Cabañes FJ, Bragulat MR, Castellá G (2010). Ochratoxin A producing species in the genus *Penicillium*. Toxins.

[4] O’Callaghan J, Caddick MX, Dobson ADW (2003). A polyketide synthase gene required for ochratoxin biosynthesis in *Aspergillus ochraceus*. Microbiology.

[5] Karolewiez A, Geisen R (2005). Cloning a part of the ochratoxin A biosynthetic gene cluster of *Penicillium nordicum* and characterization of the ochratoxin polyketide synthase gene. Syst. Appl. Microbiol..

[6] Geisen R, Schmidt-Heydt M, Karolewiez A (2006). A gene cluster of the ochratoxin A biosynthetic genes in *Penicillium*. Mycotoxin Res..

[7] Schmidt-Heydt M, Baxter E, Geisen R, Magan N (2007). Physiological relationship betweenfood preservatives, environmental factors, ochratoxin and otapksPV gene expression by *Penicillium verrucosum*. Int. J. Food Microbiol..

[8] Bacha N, Atoui A, Mathieu F, Liboz T, Lebrihi A (2009). *Aspergillus westerdijkiae* polyketide synthase gene “aoks1” is involved in the biosynthesis of ochratoxin A. Fungal Gen. Biol..

[9] Gallo A (2009). Characterisation of a pks gene which is expressed during ochratoxin A production by *Aspergillus carbonarius*. Int. J. Food Microbiol..

[10] Gallo A (2012). New insight in the ochratoxin A biosynthetic pathway by deletion of an nrps gene in *Aspergillus carbonarius*. Appl. Environ. Microbiol..

[11] Abbas A (2013). Functional characterization of the polyketide synthase gene required for ochratoxin A biosynthesis in *Penicillium verrucosum*. Int. J. Food Microbiol..

[12] Gallo A (2014). Identification and characterization of the polyketide synthase involved in ochratoxin A biosynthesis in *Aspergillus carbonarius*. Int. J. Food Microbiol..

[13] Nguyen DT (2016). Ochratoxin A production by *Penicillium thymicola*. Fungal Biol..

[14] Castellá G, Alborch L, Bragulat MR, Cabañes FJ (2015). Real time quantitative expression study of a polyketide synthase gene related to ochratoxin a biosynthesis in *Aspergillus niger*. Food Control.

[15] Gil-Serna J, Vázquez C, González-Jaén MT, Patiño B (2015). Clustered array of ochratoxin A biosynthetic genes in *Aspergillus steynii* and their expression patterns in permissive conditions. Int. J. Food Microbiol..

[16] Zhang J (2016). A polyketide synthase encoded by the gene An15g07920 is involved in the biosynthesis of ochratoxin A in *Aspergillus niger*. J. Agric. Food Chem..

[17] Nordberg H (2014). The genome portal of the Department of Energy Joint Genome Institute: 2014 updates. Nucleic Acids Res..

[18] de Vries RP (2017). Comparative genomics reveals high biological diversity and specific adaptations in the industrially and medically important fungal genus *Aspergillus*. Genome Biol..

[19] Cabañes FJ (2015). Rapid genome resequencing of an atoxigenic strain of *Aspergillus carbonarius*. Sci. Rep..

[20] Ferrara M (2016). Identification of a halogenase involved in the biosynthesis of ochratoxin A in *Aspergillus carbonarius*. Appl. Environ. Microbiol..

[21] Gerin D (2016). RNA-Seq reveals OTA-related gene transcriptional changes in *Aspergillus carbonarius*. PLoS One.

[22] Ehrlich, K. C. Genetic diversity in *Aspergillus flavus* and its implications for agriculture. Aspergillus *in the Genomic Era*. Varga, J. & Samson, R. A. (ed.) 233–247 (Wageningen Academic Publishers, The Netherlands, 2008).

[23] Abarca ML, Accesi F, Cano J, Cabañes FJ (2004). Taxonomy and significance of black aspergilli. Antonie van Leeuwenhoek.

[24] Cabañes FJ, Bragulat MR, Castellá G (2013). Characterization of nonochratoxigenic strains of *Aspergillus carbonarius* from grapes. Food Microbiol..

[25] Chang PK (2005). Sequence breakpoints in the aflatoxin biosynthesis gene cluster and flanking regions in nonaflatoxigenic *Aspergillus flavus* isolates. Fungal Genet. Biol..

[26] Andersen MR (2011). Comparative genomics of citric-acid-producing *Aspergillus niger* ATCC 1015 versus enzyme-producing CBS 513.88. Genome Res..

[27] Pel HJ (2007). Genome sequencing and analysis of the versatile cell factory *Aspergillus niger* CBS 513.88. Nat. Biotechnol..

[28] Frisvad JC (2007). Secondary metabolite profiling, growth profiles and other tools for species recognition and important *Aspergillus* mycotoxins. Stud. Mycol..

[29] Bragulat MR, Eustaquio A, Cabañes FJ (2017). Study on the presence of ochratoxin α in cultures of ochratoxigenic and non- ochratoxigenic strains of *Aspergillus carbonarius*. PloS One.

[30] Lind AL (2017). Drivers of genetic diversity in secondary metabolic gene clusters within a fungal species. PLoS Biol..

[31] Turner, G. Genomics and secondary metabolism in Aspergillus. In Aspergillus: *Molecular Biology and Genomics*. Machida, M., Gomi, K., (eds) 139–155 (Caister Academic Press: Norfolk, 2010).

[32] Yin WB (2012). An *Aspergillus nidulans* bZIP response pathway hardwired for defensive secondary metabolism operates through *aflR*. Mol. Microbiol..

[33] Han H (2016). Sequencing and functional annotation of the whole genome of the filamentous fungus *Aspergillus westerdijkiae*. BMC Genomics.

[34] Yin W, Keller NP (2011). Transcriptional elements in fungal secondary metabolism. J. Microbiol..

[35] Brakhage AA (2013). Regulation of fungal secondary metabolism. Nature Rev..

[36] Bayram O (2008). VelB/VeA/LaeA complex coordinates light signal with fungal development and secondary metabolism. Science.

[37] Bok JW, Keller NP (2004). LaeA, a regulator of secondary metabolism in *Aspergillus* spp. Eukaryot Cell..

[38] Crespo-Sempere A, Marin S, Sanchis V, Ramos AJ (2013). VeA and LaeA transcriptional factors regulate ochratoxin A biosynthesis in Aspergillus carbonarius. Int. J. Food Microbiol..

[39] Pfannenstiel BT (2017). Revitalization of a forward genetic screen identifies three new regulators of fungal secondary metabolism in the genus *Aspergillus*. Mbio.

[40] Bragulat MR, Abarca ML, Cabañes FJ (2001). An easy screening method for fungi producing ochratoxin A in pure culture. Int. J. Food Microbiol..

[41] Andrews S. FastQC: a quality control tool for high throughput sequence data. Available online at: http://www.bioinformatics.babraham.ac.uk/projects/fastqc (2010).

[42] Bolger AM, Lohse M, Usadel B (2014). Trimmomatic: A flexible trimmer for Illumina Sequence Data. Bioinformatics.

[43] Lassmann T, Hayashizaki Y, Daub CO (2011). SAMStat: monitoring biases in next generation sequencing data. Bioinformatics..

[44] Li H (2009). The Sequence alignment/map (SAM) format and SAMtools. Bioinformatics.

[45] Wysoker A., Tibbetts K., Fennell T. Picard tools version 1.90, http://picard.sourceforge.net (2013).

[46] McKenna A (2010). The Genome Analysis Toolkit: a MapReduce framework for analyzing next-generation DNA sequencing data. Genome Res..

[47] Sanseverino W (2015). Transposon insertions, structural variations, and SNPs contribute to the evolution of the melon genome. Mol. Biol. Evol..

[48] Rausch T (2012). Delly: structural variant discovery by integrated paired-end and split-read analysis. Bioinformatics.

[49] Cingolani P (2012). Using *Drosophila melanogaster* as a model for genotoxic chemical mutational studies with a new program, SnpSift. Front. Genet..

[50] Danecek P (2011). The variant call format and VCFtools. Bioinformatics.

[51] R Development Core Team. R: A language and environment for statistical computing. The R Foundation for Statistical Computing, Vienna, Austria. ISBN 3-900051-07-0, http://www.R-project.org (2011).

[52] Cingolani P (2012). A program for annotating and predicting the effects of single nucleotide polymorphisms, SnpEff: SNPs in the genome of *Drosophila melanogaster* strainw1118; iso-2; iso-3. Fly (Austin).

[53] Robinson JT (2011). Integrative genomics viewer. Nat. Biotechnol..

[54] Abyzov A, Urban AE, Snyder M, Gerstein M (2011). CNVnator: An approach to discover, genotype, and characterize typical and atypical CNVs from family and population genome sequencing. Genome Res..

[55] Yi G (2014). Genome-wide patterns of copy number variation in the diversified chicken genomes using next-generation sequencing. BMC Genomics.

[56] Bustin SA (2009). The MIQE guidelines: minimum information for publication of quantitative real-time PCR experiments. Clin. Chem..

[57] Pfaffl MV, Horgan GW, Dempfle L (2002). Relative expression software tool (REST©) for group-wise comparison and statistical analysis of relative expression results in real-time PCR. Nucleic Acids Res..

