# Supplementary material for: Genomic diversity in ochratoxigenic and non ochratoxigenic strains of Aspergillus carbonarius
Source: Sci Rep. 2018 Apr 3;8:5439. doi: 10.1038/s41598-018-23802-8 (PMC5883058; doi:10.1038/s41598-018-23802-8)
Supplement: Supplementary file 1 — Supplementary information [file 41598_2018_23802_MOESM1_ESM.pdf]

SUPPLEMENTARY INFORMATION

# Genomic diversity in ochratoxigenic and non ochratoxigenic strains of *Aspergillus carbonarius*

Gemma Castellá*[1], M. Rosa Bragulat[1], Laura Puig[1], Walter Sanseverino[2], and F. Javier Cabañes[1]

[1] Veterinary Mycology Group, Department of Animal Health and Anatomy, Universitat Autònoma de Barcelona, Bellaterra, Catalonia, Spain.

[2] Sequentia Biotech SL, Barcelona, Catalonia, Spain.

Supplementary Table 1. SNPs and DIPs analyses in genes of the hypothetical OTA cluster.

| Gene | | Variant type | Position in cDNA | Alternative allele | Strains having variant |
|---|---|---|---|---|---|
| ***AcOTApks*** | estExt_Genewise1Plus.C_120511 | missense_variant | 7695/7779 | G | A1137,A2160,A2579,A2594 |
| | estExt_Genewise1Plus.C_120511 | missense_variant | 7198/7779 | C | A1137,A2160,A2579,A2594 |
| | estExt_Genewise1Plus.C_120511 | missense_variant | 6779/7779 | C | A1137,A2579,A2594 |
| | estExt_Genewise1Plus.C_120511 | missense_variant | 6710/7779 | C | A2160 |
| | estExt_Genewise1Plus.C_120511 | missense_variant | 6435/7779 | T | A2579,A2594 |
| | estExt_Genewise1Plus.C_120511 | missense_variant | 6262/7779 | A | A2579,A2594 |
| | estExt_Genewise1Plus.C_120511 | missense_variant | 6180/7779 | T | A2579,A2594 |
| | estExt_Genewise1Plus.C_120511 | missense_variant | 6107/7779 | T | A2160 |
| | estExt_Genewise1Plus.C_120511 | missense_variant | 6058/7779 | A | A2160 |
| | estExt_Genewise1Plus.C_120511 | missense_variant | 5945/7779 | C | A1137,A2160,A2579,A2594 |
| | estExt_Genewise1Plus.C_120511 | missense_variant | 5891/7779 | T | A1137,A2160,A2579,A2594 |
| | estExt_Genewise1Plus.C_120511 | missense_variant | 5862/7779 | G | A1137,A2579,A2594 |
| | estExt_Genewise1Plus.C_120511 | missense_variant | 5762/7779 | C | A1137,A2160,A2579,A2594 |
| | estExt_Genewise1Plus.C_120511 | missense_variant | 5639/7779 | C | A2160 |
| | estExt_Genewise1Plus.C_120511 | missense_variant | 5388/7779 | A | A2160 |
| | estExt_Genewise1Plus.C_120511 | missense_variant | 5330/7779 | T | A2160 |
| | estExt_Genewise1Plus.C_120511 | missense_variant | 5133/7779 | C | A1137,A2160,A2579,A2594 |
| | estExt_Genewise1Plus.C_120511 | missense_variant | 5129/7779 | C | A2579,A2594 |
| | estExt_Genewise1Plus.C_120511 | frameshift_variant | 5029/7779 | GCCCCCC | A2579,A2594 |
| | estExt_Genewise1Plus.C_120511 | missense_variant | 5009/7779 | C | A2579,A2594 |
| | estExt_Genewise1Plus.C_120511 | stop_gained | 4822/7779 | T | A2160 |
| | estExt_Genewise1Plus.C_120511 | missense_variant | 4714/7779 | G | A2579,A2594 |
| | estExt_Genewise1Plus.C_120511 | missense_variant | 4628/7779 | T | A2160 |
| | estExt_Genewise1Plus.C_120511 | missense_variant | 4209/7779 | T | A1137,A2160,A2579,A2594 |
| | estExt_Genewise1Plus.C_120511 | missense_variant | 4140/7779 | T | A1137 |
| | estExt_Genewise1Plus.C_120511 | missense_variant | 3492/7779 | G | A1137 |
| | estExt_Genewise1Plus.C_120511 | missense_variant | 3404/7779 | T | A2160 |
| | estExt_Genewise1Plus.C_120511 | missense_variant | 3111/7779 | A | A1137,A2160,A2579,A2594 |

| | | | | | |
|---|---|---|---|---|---|
| | estExt_Genewise1Plus.C_120511 | missense_variant | 2610/7779 | T | A1137 |
| | estExt_Genewise1Plus.C_120511 | missense_variant | 2489/7779 | T | A2160,A2579,A2594 |
| | estExt_Genewise1Plus.C_120511 | missense_variant | 2273/7779 | G | A2160,A2579,A2594 |
| | estExt_Genewise1Plus.C_120511 | missense_variant | 1466/7779 | T | A1137,A2160,A2579,A2594 |
| | estExt_Genewise1Plus.C_120511 | missense_variant | 1400/7779 | C | A1137,A2160,A2579,A2594 |
| | estExt_Genewise1Plus.C_120511 | missense_variant | 1127/7779 | T | A2160,A2579,A2594 |
| | estExt_Genewise1Plus.C_120511 | missense_variant | 1031/7779 | T | A2160,A2579,A2594 |
| | estExt_Genewise1Plus.C_120511 | missense_variant | 695/7779 | C | A2160,A2579,A2594 |
| | estExt_Genewise1Plus.C_120511 | missense_variant | 368/7779 | T | A2160 |
| | | | | | |
| ***AcOTAnrps*** | estExt_Genemark1.C_120304 | missense_variant | 5624/5628 | G | A1137,A2579,A2594 |
| | estExt_Genemark1.C_120304 | missense_variant | 5450/5628 | T | A1137 |
| | estExt_Genemark1.C_120304 | missense_variant | 5370/5628 | A | A1137 |
| | estExt_Genemark1.C_120304 | missense_variant | 5359/5628 | C | A1137,A2579,A2594 |
| | estExt_Genemark1.C_120304 | missense_variant | 5347/5628 | C | A1137,A2579,A2594 |
| | estExt_Genemark1.C_120304 | missense_variant | 5237/5628 | T | A1137 |
| | estExt_Genemark1.C_120304 | missense_variant | 5231/5628 | C | A1137 |
| | estExt_Genemark1.C_120304 | missense_variant | 5219/5628 | C | A2579,A2594 |
| | estExt_Genemark1.C_120304 | missense_variant | 5180/5628 | T | A1137,A2579,A2594 |
| | estExt_Genemark1.C_120304 | missense_variant | 5161/5628 | C | A1137,A2579,A2594 |
| | estExt_Genemark1.C_120304 | missense_variant | 5159/5628 | A | A1137,A2579,A2594 |
| | estExt_Genemark1.C_120304 | missense_variant | 5098/5628 | C | A1137,A2579,A2594 |
| | estExt_Genemark1.C_120304 | missense_variant | 5078/5628 | G | A1137,A2579,A2594 |
| | estExt_Genemark1.C_120304 | missense_variant | 5051/5628 | G | A1137 |
| | estExt_Genemark1.C_120304 | missense_variant | 4871/5628 | T | A2579,A2594 |
| | estExt_Genemark1.C_120304 | missense_variant | 4807/5628 | G | A2579,A2594 |
| | estExt_Genemark1.C_120304 | missense_variant | 4645/5628 | C | A1137,A2579,A2594 |
| | estExt_Genemark1.C_120304 | missense_variant | 4121/5628 | C | A1137 |
| | estExt_Genemark1.C_120304 | missense_variant | 4027/5628 | T | A2579,A2594 |
| | estExt_Genemark1.C_120304 | missense_variant | 3988/5628 | T | A1137 |
| | estExt_Genemark1.C_120304 | missense_variant | 3949/5628 | C | A2579,A2594 |
| | estExt_Genemark1.C_120304 | missense_variant | 3923/5628 | A | A1137,A2579,A2594 |
| | estExt_Genemark1.C_120304 | missense_variant | 3905/5628 | T | A2579,A2594 |

| | | | | |
|---|---|---|---|---|
| estExt_Genemark1.C_120304 | stop_gained | 3804/5628 | C | A2579,A2594 |
| estExt_Genemark1.C_120304 | missense_variant | 3803/5628 | C | A2579,A2594 |
| estExt_Genemark1.C_120304 | missense_variant | 3766/5628 | C | A2579,A2594 |
| estExt_Genemark1.C_120304 | missense_variant | 3761/5628 | T | A2579,A2594 |
| estExt_Genemark1.C_120304 | missense_variant | 3737/5628 | T | A1137 |
| estExt_Genemark1.C_120304 | missense_variant | 3703/5628 | C | A2579,A2594 |
| estExt_Genemark1.C_120304 | missense_variant | 3427/5628 | T | A2579,A2594 |
| estExt_Genemark1.C_120304 | missense_variant | 3275/5628 | A | A2579,A2594 |
| estExt_Genemark1.C_120304 | missense_variant | 3155/5628 | T | A1137,A2579,A2594 |
| estExt_Genemark1.C_120304 | missense_variant | 3151/5628 | T | A1137 |
| estExt_Genemark1.C_120304 | missense_variant | 3121/5628 | C | A1137 |
| estExt_Genemark1.C_120304 | missense_variant | 3110/5628 | G | A1137,A2579,A2594 |
| estExt_Genemark1.C_120304 | missense_variant | 3050/5628 | G | A2579,A2594 |
| estExt_Genemark1.C_120304 | missense_variant | 3037/5628 | T | A1137 |
| estExt_Genemark1.C_120304 | missense_variant | 2998/5628 | T | A1137 |
| estExt_Genemark1.C_120304 | missense_variant | 2952/5628 | A | A1137 |
| estExt_Genemark1.C_120304 | missense_variant | 2816/5628 | T | A2579,A2594 |
| estExt_Genemark1.C_120304 | missense_variant | 2788/5628 | G | A2579,A2594 |
| estExt_Genemark1.C_120304 | missense_variant | 2749/5628 | C | A1137,A2579,A2594 |
| estExt_Genemark1.C_120304 | missense_variant | 2723/5628 | G | A1137 |
| estExt_Genemark1.C_120304 | missense_variant | 2645/5628 | A | A1137,A2579,A2594 |
| estExt_Genemark1.C_120304 | missense_variant | 2609/5628 | A | A1137,A2579,A2594 |
| estExt_Genemark1.C_120304 | missense_variant | 2608/5628 | T | A1137,A2579,A2594 |
| estExt_Genemark1.C_120304 | missense_variant | 2512/5628 | G | A2579,A2594 |
| estExt_Genemark1.C_120304 | missense_variant | 2254/5628 | T | A2579,A2594 |
| estExt_Genemark1.C_120304 | missense_variant | 2156/5628 | G | A2579,A2594 |
| estExt_Genemark1.C_120304 | missense_variant | 2135/5628 | A | A1137,A2579,A2594 |
| estExt_Genemark1.C_120304 | missense_variant | 2081/5628 | A | A2579,A2594 |
| estExt_Genemark1.C_120304 | missense_variant | 1982/5628 | C | A1137 |
| estExt_Genemark1.C_120304 | missense_variant | 1729/5628 | G | A2579,A2594 |
| estExt_Genemark1.C_120304 | missense_variant | 1342/5628 | A | A1137 |
| estExt_Genemark1.C_120304 | missense_variant | 1243/5628 | C | A1137,A2579,A2594 |
| estExt_Genemark1.C_120304 | missense_variant | 1130/5628 | T | A1137,A2579,A2594 |

| | | | | | |
|---|---|---|---|---|---|
| | estExt_Genemark1.C_120304 | missense_variant | 1048/5628 | T | A1137,A2579,A2594 |
| | estExt_Genemark1.C_120304 | missense_variant | 859/5628 | T | A2579,A2594 |
| | estExt_Genemark1.C_120304 | missense_variant | 632/5628 | A | A1137,A2579,A2594 |
| | estExt_Genemark1.C_120304 | missense_variant | 578/5628 | T | A2579,A2594 |
| | estExt_Genemark1.C_120304 | missense_variant | 410/5628 | C | A1137,A2579,A2594 |
| | estExt_Genemark1.C_120304 | missense_variant | 403/5628 | T | A1137,A2579,A2594 |
| | estExt_Genemark1.C_120304 | missense_variant | 280/5628 | T | A2579,A2594 |
| | estExt_Genemark1.C_120304 | missense_variant | 274/5628 | C | A2579,A2594 |
| | estExt_Genemark1.C_120304 | missense_variant | 227/5628 | C | A2579,A2594 |
| | estExt_Genemark1.C_120304 | missense_variant | 215/5628 | T | A1137 |
| | estExt_Genemark1.C_120304 | missense_variant | 197/5628 | C | A1137,A2579,A2594 |
| | estExt_Genemark1.C_120304 | missense_variant | 151/5628 | T | A2579,A2594 |
| | estExt_Genemark1.C_120304 | missense_variant | 127/5628 | G | A1137,A2579,A2594 |
| | estExt_Genemark1.C_120304 | missense_variant | 116/5628 | C | A2579,A2594 |
| | estExt_Genemark1.C_120304 | missense_variant | 110/5628 | A | A2579,A2594 |
| ***AcOTAhal*** | fgenesh_isotigs_kg.12_#_278_#_isotig01841 | missense_variant | 601/2382 | T | A2160 |
| ***AcOTAp450*** | estExt_fgenesh2_pm.C_120208 | missense_variant | 1275/1657 | T | A1137 |
| | estExt_fgenesh2_pm.C_120208 | missense_variant | 803/1657 | A | A1137 |
| | estExt_fgenesh2_pm.C_120208 | missense_variant | 731/1657 | T | A1137 |
| | estExt_fgenesh2_pm.C_120208 | missense_variant | 282/1657 | T | A1137 |
| | estExt_fgenesh2_pm.C_120208 | missense_variant | 53/1657 | C | A1137 |
| | estExt_fgenesh2_pm.C_120208 | missense_variant | 35/1657 | A | A1137 |
| | estExt_fgenesh2_pm.C_120208 | missense_variant | 29/1657 | C | A1137 |
| ***AcOTAbZIP*** | Genemark1.7821_g | missense_variant | 81/745 | A | A1137 |
| | Genemark1.7821_g | missense_variant | 131/745 | A | A1137 |
| | Genemark1.7821_g | missense_variant | 139/745 | C | A1137 |
| | Genemark1.7821_g | missense_variant | 421/745 | G | A1137 |
| | Genemark1.7821_g | missense_variant | 422/745 | C | A1137 |

Supplementary Table 2. Primer pairs used in RT-qPCR analysis

| Gene | Primer | Sequence (5'–3') | Concentration used | Reference |
|---|---|---|---|---|
| *AcOTAnrps* | AcNRPSF | TCGGGATAAGGGCACAAATG | 100 nM | This study |
| | AcNRPSR | CGGTCCGTTGATTCAGCAA | | |
| *AcOTApks* | RT_AcOTApks_F | CGTGTCCGATACTGTCTGTGA | 100 nM | 12 |
| | RT_AcOTApks_R | GCATGGAGTCCTCAAGAACC | | |
| *AcOTAhal* | RT_AcOTAhal_F | GAACGCCAGTAGAGGGACAG | 200 nM | 20 |
| | RT_AcOTAhal_R | ATGGAGGTGGTGTTGTTGTG | | |
| *AcOTAp450* | RT_AcOTAp450_F | GTGGTTATCCCGCCCAATAC | 200 nM | 20 |
| | RT_AcOTAp450_R | TGCCAGATTCATCCCGATAC | | |
| *AcOTAbZIP* | RT_AcOTAbZip_F | AATGGAACCAGCATTGATCTC | 200 nM | 20 |
| | RT_AcOTAbZip_R | GACCCAAGCATTCGCTCTA | | |
| β-tubulin | RT3 BT Ac_F | CAAACCGGCCAGTGTGGTA | 100 nM | 12 |
| | RT3 BT Ac_R | CGGAGGTGCCATTGTAAACA | | |
| Ubiquitin-conjugating enzyme | ubFw | CCGAAGGTCAACTTCACCAC | 200 nM | 21 |
| | ubRev | GGCATATTTGCGAGTCCATT | | |
